# Supplementary material for: Evaluation of a Florfenicol Formulation for Treating Streptococcosis and Francisellosis in Nile Tilapia (Oreochromis niloticus): A Study of Safety, Withdrawal Period and Efficacy
Source: Microorganisms. 2025 Mar 1;13(3):558. doi: 10.3390/microorganisms13030558 (PMC11944537; doi:10.3390/microorganisms13030558)
Supplement: Supplementary file 1 [file microorganisms-13-00558-s001.zip › microorganisms-3458887-supplementary.pdf]

microorganisms

MDPI

Article

Evaluation of a Florfenicol Formulation for Treating Streptococcosis and Francisellosis in Nile Tilapia (*Oreochromis niloticus*): A Study of Safety, Withdrawal Period and Efficacy

Natália Amoroso Ferrari [1], Raffaella Menegheti Mainardi [1], Mayza Brandão da Silva [1], Gabriel Diogo Guimarães [1], Marcos Letaif Gaeta [1], Francisco Eduardo Pereira Rocha [1], Tainara Oliveira da Silva [1], Alene Santos Souza [1], Bruna Cordeiro Baptista [2], João Anderson Keiti Rocha [2], Erika Fernandes Lopes Maturana [3], Daniela Dib Gonçalves [4], Nelson Maurício Lopera Barrero [5], Giovana Wingeter Di Santis [3] and Ulisses de Pádua Pereira [1,*]

1 Laboratory of Fish Bacteriology, Department of Preventive Veterinary Medicine, State University of Londrina, Londrina 86057-970, Paraná, Brazil; natalia.amoroso@uel.br (N.A.F.); raffaella.menegheti@uel.br (R.M.M.); mayza.brandao.silva@uel.br (M.B.d.S.); gabriel.diogo.guimaraes@uel.br (G.D.G.); mlgaeta@uel.br (M.L.G.); edu13rocha@gmail.com (F.E.P.R.); ttainaraoliveira.s@hotmail.com (T.O.d.S.); alenesantos47@gmail.com (A.S.S.)

2 SAN Group Biotech Brazil Ltda., Campinas 13058-009, São Paulo, Brazil; bruna.baptista@san-group.com (B.C.B.); joao.rocha@san-group.com (J.A.K.R.)

3 Laboratory of Animal Pathology, Department of Preventive Veterinary Medicine, State University of Londrina, Londrina 86057-970, Paraná, Brazil; lopesmaturana@gmail.com (E.F.L.M.); giovanaws@uel.br (G.W.D.S.)

4 Department of Preventive Veterinary Medicine and Public Health, Paranaense University, Umuarama 87502-210, Paraná, Brazil; danieladib@unipar.br (D.D.G.)

5 Department of Animal Science, State University of Londrina, Londrina 86057-970, Paraná, Brazil; nmlopera@uel.br (N.M.L.B.)

* Correspondence: upaduapereira@uel.br; Tel.: +55-43-3371-4765

microorganisms

MDPI

Supplementary Table S1. Scoring system for histopathological analysis of Nile tilapia during and after treatment with florfenicol.

Organ	Parameter	Score
Stomach (0 to 3 total score)	Inflammation, congestion, necrosis	0 – Absent
		1 – Rare/Mild
		2 – Moderate
		3 – Accentuated
Spleen (0 to 3 total score)	Congestion, necrosis, pigmented macrophage	0 - Absent
		1 – Rare/Mild
		2 – Moderate
		3 – Accentuated
Liver (1 to 3 total score)	Intracellular accumulations	1 - Abundant glycogen storage
		2 - Slight to moderate reduction in glycogen storage
		3 - Marked reduction in glycogen storage and/or moderate to marked accumulation of lipids
	Inflammation, congestion, necrosis, pigmented macrophage	0 - Absent
		1 – Rare/Mild
		2 – Moderate
		3 – Accentuated
